# The neurophysiological basis of stress and anxiety - comparing neuronal diversity in the bed nucleus of the stria terminalis (BNST) across species

**DOI:** 10.3389/fncel.2023.1225758

**Published:** 2023-08-30

**Authors:** Yana van de Poll, Yasmin Cras, Tommas J. Ellender

**Affiliations:** ^1^Department of Biomedical Sciences, University of Antwerp, Antwerp, Belgium; ^2^Department of Pharmacology, University of Oxford, Oxford, United Kingdom

**Keywords:** bed nucleus of the stria terminalis (BNST), electrophysiology, neurpeptides, cross-species, rodents, macaque, human

## Abstract

The bed nucleus of the stria terminalis (BNST), as part of the extended amygdala, has become a region of increasing interest regarding its role in numerous human stress-related psychiatric diseases, including post-traumatic stress disorder and generalized anxiety disorder amongst others. The BNST is a sexually dimorphic and highly complex structure as already evident by its anatomy consisting of 11 to 18 distinct sub-nuclei in rodents. Located in the ventral forebrain, the BNST is anatomically and functionally connected to many other limbic structures, including the amygdala, hypothalamic nuclei, basal ganglia, and hippocampus. Given this extensive connectivity, the BNST is thought to play a central and critical role in the integration of information on hedonic-valence, mood, arousal states, processing emotional information, and in general shape motivated and stress/anxiety-related behavior. Regarding its role in regulating stress and anxiety behavior the anterolateral group of the BNST (BNST_ALG_) has been extensively studied and contains a wide variety of neurons that differ in their electrophysiological properties, morphology, spatial organization, neuropeptidergic content and input and output synaptic organization which shape their activity and function. In addition to this great diversity, further species-specific differences are evident on multiple levels. For example, classic studies performed in adult rat brain identified three distinct neuron types *(Type I-III)* based on their electrophysiological properties and ion channel expression. Whilst similar neurons have been identified in other animal species, such as mice and non-human primates such as macaques, cross-species comparisons have revealed intriguing differences such as their comparative prevalence in the BNST_ALG_ as well as their electrophysiological and morphological properties, amongst other differences. Given this tremendous complexity on multiple levels, the comprehensive elucidation of the BNST_ALG_ circuitry and its role in regulating stress/anxiety-related behavior is a major challenge. In the present Review we bring together and highlight the key differences in BNST_ALG_ structure, functional connectivity, the electrophysiological and morphological properties, and neuropeptidergic profiles of BNST_ALG_ neurons between species with the aim to facilitate future studies of this important nucleus in relation to human disease.

## Introduction

Rodents, specifically rats (*Rattus norvegicus*) and mice (*Mus musculus*), are preferred animal models in the field of biomedical sciences because of their many anatomical, physiological, and genetic similarities to humans. Specifically in the field of neuroscience, rats have often been selected to study activity within complex neuronal circuits especially in relation to behavior. However, in the last few decades a shift has occurred as a result of the development of transgenic mice, with studies using mice predominating over those using rats (Ellenbroek and Youn, 2016). This transition and the generation of many transgenic mouse lines has greatly aided progress in the field of neuroscience. However, it does raise important questions whether and to what extent it is possible to generalize between mouse and rat data, notwithstanding the larger questions also how this research translates to humans. Indeed, it is important to note that, apart from well-known evolutionary divergences, in some instances clear and striking differences have been found at the cellular, molecular, anatomical, and behavioral level. For example, although certain key structures that are embedded in the brain’s stress and anxiety circuitry would be expected to be conserved across mammalian species, and indeed this is true for certain aspects, species-specific differences are evident on several levels.

As part of the brain’s stress and anxiety circuitry, the bed nucleus of the stria terminalis (BNST), has become of increasing interest with regard to its role in several human psychiatric disorders, including post-traumatic stress disorder (PTSD), generalized anxiety disorder (GAD), social anxiety, and addiction (Ahearn et al., 2007; Buff et al., 2017; Clauss et al., 2019; Feola et al., 2023). This complex brain structure can be divided in multiple sub-regions and, in rodents at least, is thought to consist of 11 to 18 different sub-nuclei. Located in the basal forebrain, anterior to the hypothalamus and adjacent to the amygdala and striatum, the BNST receives and sends projections onto a variety of limbic brain structures. Given this connectivity, the BNST, as part of the extended amygdala, is thought to maintain online information about mood, arousal, hedonic-valence, and sensory stimuli, shaping and regulating not only motivated and stress/anxiety-related behavior, but also social behavior. In addition to its role in stress and anxiety behavior, the BNST is also thought to play a role in monitoring sustained threats and regulating avoidance behavior. Maybe unsurprisingly considering these many ascribed functional roles the complexity and diversity in BNST neuronal cell types is vast, with many of them exhibiting complex spatial organizations, expressing diverse neurochemicals and having distinct input and output synaptic organizations and neurophysiological properties. Classic studies performed in adult rat brains have identified three distinct BNST neuron types *(Type I, II, and III)*, based on their electrophysiological properties and ion channel expression. Although neurons with similar electrophysiological properties have been identified in other animal species, including mice and non-human primate, cross-species comparisons reveal intriguing differences, such as their comparative prevalence in the BNST as well as their electrophysiological and morphological characteristics, with detailed information on many other important features such as their connectivity or neurochemical content currently lacking.

The aim of this Review is to provide an assessment of the similarities and differences in the BNST amongst species and consider how these differences, including anatomical structure, functional connectivity, neuropeptidergic content, and electrophysiological and morphological properties of neurons, may impact our understanding of these neuronal circuits and their roles in regulating stress/anxiety-related behavior. The focus of this Review will mostly be on the dorsal BNST_ALG_ (and in part the amBNST) and will discuss its role in regulating anxiety and compare mouse, rat, human and non-human primate data. We would like to refer to excellent reviews on the role of the BNST in social behavior (Flanigan and Kash, 2022), disorders (Lebow and Chen, 2016), in humans (Avery et al., 2016), in sleep-wake and emotional arousal (Giardino and Pomrenze, 2021), transcriptome and cell types (Ortiz-Juza et al., 2021), addiction (Stamatakis et al., 2014) and regulation of anxiety- and stress-related behaviors (Avery et al., 2016; Gungor and Paré, 2016; Lebow and Chen, 2016; Klumpers et al., 2017; Ch’ng et al., 2018).

## Anatomy and functional connectivity in the BNST

### Anatomy

**FIGURE 1:**
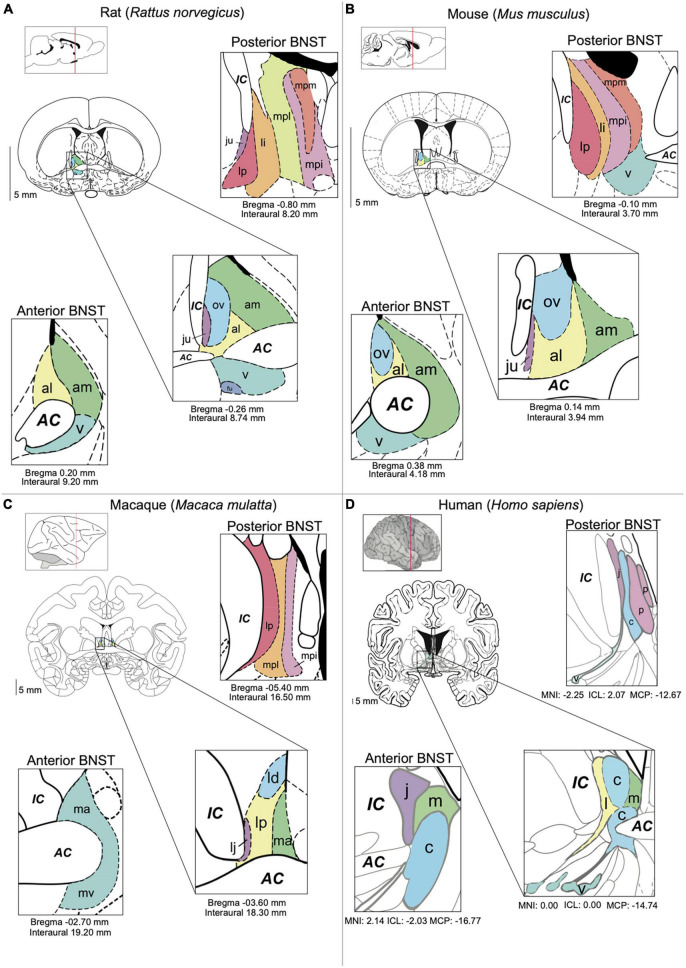
BNST anatomy and nomenclature in different species. **(A)** Main nuclei of the BNST in the rat [Paxinos (1995) The Rat Brain in Stereotaxic Coordinates, 2nd edition]. **(B)** Main nuclei of the BNST in the mouse. **(C)** The BNST in the macaque (Paxinos et al., 2000). The Macaque Brain in Stereotaxic Coordinates, 2nd edn.). **(D)** Main nuclei of the BNST in the human [Mai (2016). Atlas of the Human Brain. 4th Ed.]. Subnuclei are colored based on their anatomic similarities. AC = anterior commissure, al = anterolateral BNST, am = anteromedial BSNT, c = central BNST, IC = internal capsule, ju = juxtacapsular BNST, l = lateral BNST, ld = laterodorsal BNST, lp = lateral posterior BNST, li = lateral intermediate BNST, m = medial BNST, ma = medial anterior BNST, mpi = medial posterointermediate BNST, mpl = medial posterolateral BNST, mpm = medial posteromedial BNST, mv = medioventral BNST, ov = oval BNST, p = posterior BNST, v = ventral BNST.

The BNST is a diverse forebrain region made up of around 11 to 18 subnuclei in rodents. Described as a component of the extended amygdala, which comprises the BNST, the central and medial amygdala (CeA and MeA, respectively) as well as parts of the nucleus accumbens (NAc) (de Olmos and Heimer, 1999), the BNST is located ventral to the lateral ventricles, medial to the internal capsule and surrounding the anterior commissure (Figure 1). First defined by Johnston (1923), at that time named the bed of the stria terminalis, the definition and subdivision of this region has undergone major changes throughout the years and remains unstandardized. Early anatomists further divided the BNST into medial and lateral subdivisions, based on their respective location to the midline and internal capsule. Later studies separated the BNST relative to its anterior and posterior axis, based on separation by fibers of the stria terminalis as well as neuronal morphology and neuropeptidergic content (De Olmos and Ingram, 1972; Krettek and Price, 1978; Weller and Smith, 1982; Dong et al., 2001b; Egli and Winder, 2003). Within the last decade a combination of the medial/lateral and anterior/posterior division has been commonly adopted. Moreover, in most mammalian species these nuclei within the anterior and posterior region are now commonly further subdivided. These consist for the anterior BNST in rodents of the anterolateral, anteromedial, oval, juxtacapsular, and fusiform nuclei (alBNST, amBNST, ovBNST, juBNST, and fuBNST, respectively), whilst the posterior BNST is comprised of the principal, rhomboid, magnocellular, dorsomedial, ventral, and dorsal nuclei (prBNST, rhBNST, mgBNST, dmBNST, vBNST, and dBNST, respectively). Several of these subnuclei in the anterior region are often grouped together to form the anterolateral group (BNST_ALG_), consisting of alBNST, ovBNST, juBNST, and fuBNST and this is the predominant region considered in this Review (Figure 1). Even though general functions of the BNST appear largely conserved across species, many of these further anatomical subdivisions in non-human primates and humans have not been extensively described, most likely due to limitations in techniques used, such as lower spatial resolution with functional and resting state magnetic resonance imaging (fMRI) as compared to the availability of high-resolution methods such as *in vivo* imaging and electrophysiology in rodents. For example, the majority of studies in humans only describe lateral (BNSTl), central (BNSTc), juxtacapsular (BNSTj), medial (BNSTm), ventral (BNSTv) and posterior (BNSTp) subdivisions (Walter et al., 1991). Even though the human BNST is divided into fewer subnuclei, the overall BNST is seen to be relatively bigger and more developed as compared to rodents, (Avery et al., 2014). In non-human primates such as the macaque, subdivisions appear similar to those observed in rodents. Here, the BNST is divided in the laterodorsal (BNSTld), latero juxta capsular (BNSTlj), medial anterior (BNSTma), ventral (BNSTmv), and lateral posterior (BNSTlp) nucleus (Paxinos et al., 2000).

### Functional connectivity

A major feature of the BNST is its extensive connectivity with downstream brain targets that are known to be embedded within stress- and anxiety-related neuronal circuits. By connecting to these targets, the BNST plays a major role in mediating both autonomic and behavioral reactions to stress and anxiety (Walker and Davis, 1997). Lesion studies in rats, and later optogenetic studies in mice (Kim et al., 2013), have demonstrated that activity in the anterior BNST can be either anxiogenic (increasing anxiety) or anxiolytic (reducing anxiety) depending on the lesion site and is crucial for regulating sustained responding to, or anticipation of, an aversive event, also known as anticipatory anxiety behavior (Hammack et al., 2004, 2015; Bangasser et al., 2005; Resstel et al., 2008). In contrast to those found anterior the subnuclei located in the posterior BNST are believed to be more involved in reproductive and autonomic behaviors (Dong and Swanson, 2003, 2006). However, some functions do appear to cross boundaries to some extent [e.g., rodent feeding and maternal behavior are in part regulated by the anterior as well as posterior BSNT (Flanigan and Kash, 2022)], likely as a result of many subnuclei within the BNST communicating with each other through local connections (Dong et al., 2000, 2001a; Dong and Swanson, 2004; Larriva-Sahd, 2006). These local connections will not be discussed further in detail here. Instead, this Review will predominantly focus on the cellular and circuit properties of the dorsal BNST_ALG_ and its long-range inputs and outputs. Many connectivity studies regarding BNST_ALG_ subnuclei were performed in the early 2,000 s in male rats and therefore much of the knowledge of the BNST connectivity therefore represents that of the male rat brain (Dong et al., 2000, 2001a,b; Dong and Swanson, 2003, 2004, 2006; Dabrowska et al., 2013). More recently, connectivity studies have also been performed in mice and in macaques, using more modern viral tracing approaches (deCampo and Fudge, 2013; Smith et al., 2016; Oler et al., 2017; Sun et al., 2023) and functional MRI (fMRI) or resting-state MRI (rsMRI) in humans (Avery et al., 2014; Krüger et al., 2015; Hofmann and Straube, 2019). Many of these studies, as discussed below, have provided insight into differences in structural and functional connectivity between species. Moreover, the rodent studies in the BNST over the past two decades have also provided insight and guided research about its potential role in human function and psychopathology (Alvarez et al., 2011; Grupe et al., 2012; Somerville et al., 2012; Brenton et al., 2014; Herrmann et al., 2016), including abnormal fear and anxiety manifested as anxiety-related psychiatric disorders such as PTSD, GAD, and social anxiety (Ahearn et al., 2007; Buff et al., 2017; Feola et al., 2023). Multiple studies have also suggested an important role for the human BNST in driving individual differences in emotional responding (e.g., an anxious temperament). Indeed, the BNST shows differences in size, connectivity, and function in men compared to women and this sexual dimorphism may (in part) explain higher prevalence of anxiety disorders in women (Zhou et al., 1995; Chung et al., 2002; Flook et al., 2020). Next, we provide an overview of the major connections of the BNST with other brain structures, linking the connectivity to its role in stress, anxiety and fear and as outlined above predominantly focusing on (functional) connectivity of the dorsal BNST_ALG_ in rodents and anterior BNST in humans and non-human primates (Table 1).

**TABLE 1:** Overview of the input-output connectivity patterns and the chemoarchitecture of the BNST_ALG_.

	Rat *(Rattus norvegicus)*	Mouse *(Mus musculus)*	Macaque *(Macaca mulatta)*	Human *(Homo sapiens)*
BNST subnucleus	alBNST (Allen et al., 1983; Swanson et al., 1983; Chronwall et al., 1985; Fallon and Leslie, 1986; Gray and Magnuson, 1987, 1992; Ju et al., 1989; Poulin et al., 2009) 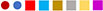 ovBNST (Allen et al., 1983; Swanson et al., 1983; Chronwall et al., 1985; Fallon and Leslie, 1986; Gray and Magnuson, 1987, 1992; Ju et al., 1989; Poulin et al., 2009) 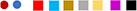 juBNST (Allen et al., 1983; Swanson et al., 1983; Chronwall et al., 1985; Fallon and Leslie, 1986; Gray and Magnuson, 1987, 1992; Ju et al., 1989; Poulin et al., 2009) 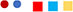 amBNST (Allen et al., 1983; Swanson et al., 1983; Chronwall et al., 1985; Fallon and Leslie, 1986; Gray and Magnuson, 1987, 1992; Ju et al., 1989; Poulin et al., 2009) 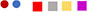	alBNST (Nguyen et al., 2016; Ortiz-Juza et al., 2021) 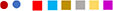 ovBNST (Nguyen et al., 2016; Smith et al., 2016; Ortiz-Juza et al., 2021) 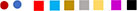 juBNST (Nguyen et al., 2016) 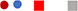 amBNST (Pleil et al., 2012; Nguyen et al., 2016; Ortiz-Juza et al., 2021) 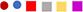	BNSTlp (deCampo and Fudge, 2013; Fudge et al., 2022) 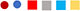 BNSTld (deCampo and Fudge, 2013; Fudge et al., 2022) 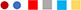 BNSTlj (deCampo and Fudge, 2013; Fudge et al., 2022) 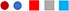 BNSTma	BNSTc (Walter et al., 1991) 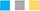 BNSTl (Adrian et al., 1983; Walter et al., 1991) 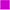 BNSTlj BNSTm (Walter et al., 1991) 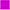
Input	**Cerebral Cortex:** – mPFC (Radley et al., 2009) – Insula (Dong et al., 2001a) – Infralimbic Cortex (Vertes, 2004) – Prelimbic Cortex (Vertes, 2004) **Amygdala:** – CeA (Myers et al., 2014) – MeA (Dong et al., 2001b) – BLA (Krettek and Price, 1978) **Hippocampus:** – Subiculum (Dong et al., 2001a) **Hypothalamus:** – PVN (Berk and Finkelstein, 1982) – MPN (Myers et al., 2014) – VMH (Myers et al., 2014) – mPOA (Myers et al., 2014) **Thalamus:** – PVT (Dong et al., 2001a) **Midbrain/Hindbrain:** – PBN (Dong et al., 2001a) – LC (Dong et al., 2001a) – VTA (Dong et al., 2001a; Hasue and Shammah-Lagnado, 2002) – NST (Dong et al., 2001a) – DR (Azmitia and Segal, 1978; Petit et al., 1995) – LS (Myers et al., 2014) **Basal ganglia:** – NAc (Myers et al., 2014) – SNc (Hasue and Shammah-Lagnado, 2002)	**Cerebral Cortex:** – Insula (Sun et al., 2023) – Prelimbic cortex (Sun et al., 2023) – Anterior cingulate cortex (Sun et al., 2023) – Piriform cortex (Sun et al., 2023) – Entorhinal cortex (Sun et al., 2023) – Perirhinal cortex (Sun et al., 2023) **Amygdala:** – CeA (Sun et al., 2023) – MeA (Sun et al., 2023) – BLA (Vantrease et al., 2022; Sun et al., 2023) **Hippocampus:** – Subiculum (Sun et al., 2023) **Hypothalamus:** – POA (Sun et al., 2023) – TMN (Lin et al., 2023) **Thalamus:** – Anteromedial (Sun et al., 2023) **Midbrain/Hindbrain:** – PAG (Sun et al., 2023) – LS (Sun et al., 2023) – DR (Sun et al., 2023) **Basal ganglia:** – pDMS (Lu et al., 2021)	**Cerebral Cortex:** – OFC (Fox et al., 2010) – ? **Amygdala:** (Novotny, 1977) – CeA (deCampo and Fudge, 2013; Oler et al., 2017) – BLA (deCampo and Fudge, 2013) – SLEA (Oler et al., 2017) **Hippocampus:** – Subiculum (Berry et al., 2022) **Hypothalamus:** – ? **Thalamus:** – ? **Midbrain/Hindbrain:** – VTA (Fudge and Haber, 2001) – ? **Basal Ganglia:** – NAc (Kalin et al., 2005) – ?	**Cerebral Cortex:** – mPFC (Krüger et al., 2015; Torrisi et al., 2015) – dlPFC (Krüger et al., 2015; Torrisi et al., 2015) – vmPFC (Motzkin et al., 2015) – OFC (Krüger et al., 2015) – Precuneus (Torrisi et al., 2015) – Insula (Flook et al., 2020) **Amygdala** (Avery et al., 2014): CeA (Krüger et al., 2015; Gungor and Paré, 2016; Shackman and Fox, 2016; Brinkmann et al., 2018; Hofmann and Straube, 2019, 2021)- BLA (Hofmann and Straube, 2019, 2021) – SLEA (Hofmann and Straube, 2019) **Hippocampus** (Avery et al., 2014): – Subiculum (Berry et al., 2022) **Hypothalamus** – PVN (Krüger et al., 2015) **Thalamus** (Avery et al., 2014): – IL/CM midline (Torrisi et al., 2015) **Midbrain/hindbrain:** – DR (Torrisi et al., 2015) – PAG (Torrisi et al., 2015) – Habenula (Torrisi et al., 2015) **Basal ganglia:** – Caudate nucleus (Torrisi et al., 2015) – Putamen (Avery et al., 2014) – NAc (Torrisi et al., 2015) *Note: *Human input-output is mostly based on magnetic resonance imaging (MRI) data. In particular, functional/resting state connectivity patterns.*
Output	**Cerebral Cortex:** – Prelimbic cortex (Dabrowska et al., 2016) 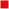 Prelimbic cortex (Dabrowska et al., 2016) 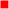 – Habenula (Dabrowska et al., 2016) **Amygdala:** – CeA (Dong et al., 2001a; Dong and Swanson, 2004; Dabrowska et al., 2016) 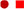 – BLA (Dong et al., 2001a; Dabrowska et al., 2016; Leitermann et al., 2016) 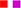 – MeA (Dabrowska et al., 2016) 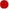 **Hypothalamus:** – PVN (Dong et al., 2001a; Dabrowska et al., 2016) 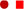 – LH (Dong et al., 2001a) 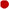 **Thalamus** – Subthalamic nucleus (Dong et al., 2001a) – Midline nuclei (Dong et al., 2001a) – PVT (Dabrowska et al., 2016; Brinkmann et al., 2018) 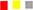 **Midbrain/Hindbrain:** – PAG (Gray and Magnuson, 1987; Dong and Swanson, 2004, 2006; Dabrowska et al., 2016) 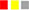 – VTA (Gray and Magnuson, 1987; Dong et al., 2001a; Dabrowska et al., 2016) 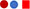 – PBN (Dong et al., 2001a; Brinkmann et al., 2018) 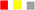 – Zona incerta (Dong et al., 2001a) – DR (Dong et al., 2001a; Dabrowska et al., 2016) 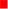 – Reticular area (Dong et al., 2001a) – Lateral Tegmental nucleus (Dong et al., 2001a) – Red Nucleus (Dabrowska et al., 2016) 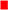 – Nucleus of the solitary tract – Substantia innominata (Dong et al., 2001a; Brinkmann et al., 2018) 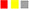 **Basal Ganglia:** – NAc (Dabrowska et al., 2016) 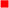 – Globus Pallidus (Dong et al., 2001a) – Septal Nucleus (Ju et al., 1989) – SNc (Dong et al., 2001a; Dabrowska et al., 2016) 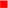	**Cerebral cortex:** – Prelimbic cortex (Dabrowska et al., 2016) **Amygdala:** – MeA (Dabrowska et al., 2016; Bruzsik et al., 2021) 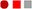 – CeA (Dabrowska et al., 2016; Bruzsik et al., 2021) 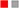 – BLA (Dabrowska et al., 2016; Bruzsik et al., 2021) 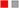 **Thalamus**: – Parasubthalamic nucleus (Lin et al., 2023) 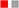 **Hypothalamus:** – PVN (Barbier et al., 2021) 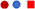 – DMH (Barbier et al., 2021) – ARH (Barbier et al., 2021) – LH (Barbier et al., 2021; Bruzsik et al., 2021) 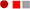 **Midbrain/Hindbrain:** – VTA (Kudo et al., 2012; Fellinger et al., 2021; Miura et al., 2022) 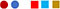 – PAG (Dabrowska et al., 2016; Bruzsik et al., 2021) 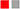 – PBN (Dabrowska et al., 2016) PBN (Dabrowska et al., 2016) – DR (Dabrowska et al., 2016; Bruzsik et al., 2021) 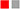 – Red Nucleus (Dabrowska et al., 2016) 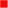 – Nucleus of the solitary tract (Dabrowska et al., 2016) – Substantia innominata (Bruzsik et al., 2021) 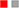 – TMN (Bruzsik et al., 2021) 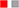 – Locus Coeruleus (Bruzsik et al., 2021) 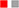 **Basal Ganglia:** – Dorsal striatum (Smith et al., 2016; Lu et al., 2021) 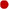 – NAc (Dabrowska et al., 2016; Xiao et al., 2021) 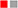 – SNc (Smith et al., 2016; Bruzsik et al., 2021) 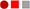 –	**Cerebral Cortex:** – OFC (Fox et al., 2018) – ? **Amygdala:** (Novotny, 1977) – SLEA (Oler et al., 2017) – CeA **Hypothalamus:** – ? **Thalamus**: – ? Midbrain/hindbrain – VTA (Fudge et al., 2017) **Basal Ganglia:** – Ventromedial striatum (Fudge et al., 2017) – NAc (Kalin et al., 2005)	**Cerebral Cortex:** – mPFC, dlPFC (Torrisi et al., 2015) – Precuneus (Torrisi et al., 2015) **Amygdala:** (Avery et al., 2014; Klumpers et al., 2017) – CeA (Gungor and Paré, 2016; Shackman and Fox, 2016; Brinkmann et al., 2018; Hofmann and Straube, 2019) – SLEA (Hofmann and Straube, 2019) **Hippocampus** (Avery et al., 2014) **Hypothalamus:** – PVN (Krüger et al., 2015; Hofmann and Straube, 2019) **Thalamus** (Avery et al., 2014): – CM midline (Torrisi et al., 2015) **Midbrain/Hindbrain:** – DR (Torrisi et al., 2015) – PAG (Torrisi et al., 2015) – Habenula (Torrisi et al., 2015) **Basal Ganglia:** – Caudate nucleus (Torrisi et al., 2015) – Putamen (Avery et al., 2014) – NAc (Torrisi et al., 2015)
Behavioural function and dysfunction	– Stress induced nociception (Tran et al., 2012) – Autonomic stress response (modulating ACTH release) (Dong et al., 2001a) – Contextual, cued, and unpredictable threat processing (Walker et al., 2009; Urien and Bauer, 2022) – (Conditioned) anxiety-like behaviour (Sahuque et al., 2006; Sink et al., 2013; Sherman et al., 2022) – Modulation of behavioural despair (Pezük et al., 2006) – Modulating stress-induced changes in sensorimotor gating (Rajbhandari and Bakshi, 2020) – Modulating locomotor, orofacial and ingestive behaviour (Dong et al., 2001a) – PTSD (Rodríguez-Sierra et al., 2016) – Chronic Stress (Normandeau et al., 2018) – Alcohol/drugs-motivated behaviour (Epping-Jordan et al., 1998; Eiler et al., 2003) – Anxiety like behaviour during withdrawal (Huang et al., 2010)	(Acute) pain related behaviors (Myers et al., 2014; Fang et al., 2023) – Arousal (Ju et al., 1989) – Conditioned anxiety/ contextual fear/ sustained anxiety (Sullivan et al., 2004; Vantrease et al., 2022) – Sustained anxiety/threat anticipation/ threat processing (Fellinger et al., 2021) – Pavlovian fear and reward behaviour/learning (Bruzsik et al., 2021; Kaouane et al., 2021) – Regulation of food intake (Wang et al., 2019) – Alcohol- and drug-motivated behaviour (Silberman et al., 2013)	– Anxiety related responses (freezing behaviour) (Kalin et al., 2005) – Threat anticipation/evaluation of threatening stimuli (Hoffman et al., 2007) – Behavioural inhibition (Fox et al., 2010) – Social/affiliative behaviour: aggression, mating behaviour, maternal care, social interaction (Jacobs et al., 2023)	– Sustained anxiety/threat anticipa tion/threat processing (Davis et al., 2010; Alvarez et al., 2011; Grupe et al., 2012; Somerville et al., 2013; Herrmann et al., 2016; Brinkmann et al., 2018; Camilo et al., 2022) – Phasic threat processing (Gungor and Paré, 2016; Shackman and Fox, 2016; Klumpers et al., 2017; Brinkmann et al., 2018; Koller et al., 2019) – Anxious temperament (Fox et al., 2018) – PTSD (Ahearn et al., 2007; Brinkmann et al., 2017; Awasthi et al., 2020; Feola et al., 2023) – Generalized Anxiety disorder (Avery et al., 2016; Buff et al., 2017; Brinkmann et al., 2018) – Social Anxiety (Avery et al., 2016; Clauss, 2019; Clauss et al., 2019) – Affective disorders (Fox et al., 2010)

alBNST, anterolateral BNST; amBNST, anteromedial BNST; BNSTc, central BNST; BNSTl, lateral BNST; BNSTld, laterodorsal BNST; BNSTlj, lateral juxtacapsular BNST; BNSTlp, lateral posterior BNST; BNSTma, medial anterior BNST; BNSTm, medial BNST; juBNST, juxtacapsular BNST; ovBNST, oval BNST; BLA, basolateral amygdala; CeA, central amygdala; CM, centromedian nucleus; dlPFC, dorsolateral prefrontal cortex; DR, dorsal raphe; LC, locus coeruleus; LS, lateral septum; MeA, medial amygdala; mPFC, medial prefrontal cortex; MPN, median preoptic nucleus; mPOA, median preoptic area; NAc, nucleus accumbens; NST, solitary nucleus; PBN, parabrachial nucleus; PVN, paraventricular nucleus; SLEA, sublenticular extended amygdala; SNc, substantia nigra pars compacta; VMH, ventromedial hypothalamus; VTA, ventral tegmental area; ACTH, adrenocorticotropic hormone; CRF, corticotropin releasing factor; DYN, dynorphin; ENK, enkephalin; GABA, gamma-aminobutyric acid; NPY, neuropeptide Y; NT, neurotensin; PKC, protein kinase C type delta; PTSD, post-traumatic stress disorder; SOM, somatostatin.

NEUROTRANSMITTERS

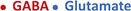

NEUROPEPTIDES/ENZYMES

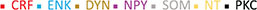

### Long-range BNST input & output

Even though the long-range outputs of the different BNST_ALG_ subnuclei are diverse and extensive, there are similar and principal patterns of targeted brain regions observed amongst species (Table 1). Most of these BNST connections are reciprocal with target brain regions sending projections back to BNST, i.e., inputs to the BNST_ALG_ in rodents are similar as its output regions. Here only the major outputs related to functions in stress and anxiety will be discussed and include specific regions of the cerebral cortex, amygdala, hypothalamus and thalamus, basal ganglia, and autonomic brain centers in the midbrain/hindbrain. These outputs consist of both GABAergic and glutamatergic projections and in rodents, follow two main tracts; the stria terminalis and the ansa peduncularis (Dong et al., 2001a). Of all these input sites the basolateral amygdala (BLA), regions of the prefrontal cortex (PFC), hippocampus, and the paraventricular nucleus (PVN) of the thalamus, mainly provide glutamatergic inputs (Canteras and Swanson, 1992; Myers et al., 2014), whilst the central amygdala (CeA), medial amygdala (MeA), and NAc mainly provide GABAergic inputs (Myers et al., 2014). The lateral hypothalamus, including the PVN, provides both glutamatergic and GABAergic input (Berk and Finkelstein, 1982; Myers et al., 2014). This diverse pool of neurotransmitters together with released peptides shape BNST activity (Canteras and Swanson, 1992; Dong et al., 2001b; Sun et al., 2023). In addition, the BNST is also innervated by many neuromodulatory systems, such as dopamine, serotonin, and histamine amongst others (Garcia-Garcia et al., 2018; Gyawali et al., 2023; Lin et al., 2023), which provide a further regulation of BNST activity, but these neuromodulators will not be discussed in detail in this Review.

### Cerebral cortex

A major and important dorsal BNST_ALG_ reciprocal connection is with the cortex and in particular the PFC. Indeed, in rodents the prelimbic and anterior cingulate cortex provide the largest projections (Dong et al., 2001a; Vertes, 2004; Radley et al., 2009; Sun et al., 2023). The prelimbic cortex is thought to be involved in regulating fear expression amongst regulation of other emotions (Sun et al., 2023), while the anterior cingulate cortex is more involved in regulating emotions and cognitive control (Sun et al., 2023). Other connections from PFC include those from infralimbic regions (Reisiger et al., 2014; Glangetas et al., 2017). Even though the BNST is larger (in proportion to brain size) and more developed in humans, rs/fMRI data seems to support a high level of conservation of inputs across species with humans exhibiting some new and stronger connections with some cortical regions (Avery et al., 2014). An interesting connection seen in both rodent and human BNST is with the insular cortex (Dong et al., 2001a; Avery et al., 2014; Flook et al., 2020; Sun et al., 2023) which is thought to regulate BNST activity in relation to affective behavior (Luchsinger et al., 2021; Glangetas, 2022). Interesting is this regard is the presence of so-called Von Economo or spindle cells which are found in high numbers in insular cortex and other anterior limbic areas in humans but not in many other primates or species, potentially providing a unique input and regulation of BNST in humans only (Seeley et al., 2012; Table 1).

### Amygdala

A second major connected region is the amygdalar complex, which is comprised of 13 nuclei, of which the centro-medial complex (CeM) is highly interconnected with the BNST_ALG_ and are able to regulate sustained fear and anxiety responses. Of these amygdalar nuclei the central CeA is the major output center and is specialized in autonomic responses via its connections to various brainstem nuclei, whilst the medial MeA is more specialized in pheromone-induced responses (Dong et al., 2001b). Of all the amygdalar nuclei, the CeA receives the largest input from the rodent BNST_ALG_ (Dong et al., 2000, 2001a; Dong and Swanson, 2004). The MeA is less densely innervated by the BNST_ALG_, as it is more important in initiating autonomic responses (Dong et al., 2000; Kim et al., 2013; Nguyen et al., 2016). BNST connectivity with many of the nuclei of the amygdala has been shown to be largely reciprocal (Table 1). Indeed, in both macaques and humans, reciprocal connections between the amygdala to the BNST have been shown to be conserved (Oler et al., 2012; deCampo and Fudge, 2013; Avery et al., 2014), with the CeA having the strongest reciprocal connection in humans (Avery et al., 2014). The projections originating in the CeA are GABAergic and mainly target the ovBNST with other nuclei of the BNST_ALG_ receiving less dense innervation (Dong et al., 2001b; Gungor and Paré, 2016). These GABAergic projections are thought to reduce the overall inhibitory output of the BNST_ALG_ nuclei. Other nuclei of the amygdala, such as the BLA, MeA, and cortical nucleus, also project to the BNST_ALG_ (Sun et al., 2023) with the main difference being that these nuclei mostly provide excitatory glutamatergic input and in general have the effect of increasing inhibitory output of the BNST_ALG_. Although early findings suggested the ovBNST is the only nucleus in the BNST_ALG_ not receiving input from the BLA (Dong et al., 2001b) more recent viral tracing approaches have revealed the presence of synaptic connections (Sun et al., 2023). Moreover, functional and resting-state connectivity between the amygdala and anterior BNST has been extensively studied in relation to PTSD and GAD in humans. Patients with PTSD have been shown to have increased BNST activation and display stronger BNST and amygdala functional connectivity with multiple fear and anxiety regions, including the hypothalamus, hippocampus, insula, and ventromedial prefrontal cortex (vmPFC) as measured by fMRI (Feola et al., 2023). A rsMRI study by Buff et al. (2017) revealed that GAD patients display an increased phasic amygdala activity to onset of threat anticipation and with elevated sustained BNST activity that is delayed relative to the onset of threat anticipation. Moreover, recent rsfMRI data suggested that less BNST self-inhibition and more inhibition from the centromedial amygdala is a common pattern related to higher anxiety (Hofmann and Straube, 2019).

### Hypothalamus

The hypothalamus is an important region for the coordination of neuroendocrine functions and is heavily targeted by the BNST_ALG_ subnuclei. The paraventricular nucleus (PVN), a subnucleus of the hypothalamus, is one of the major autonomic control centers of the brain and densely targeted by outputs from the BNST_ALG_ in rodents and the anterior BNST in humans (Dong et al., 2001a; Dong and Swanson, 2004; Avery et al., 2014; Barbier et al., 2021). Via corticotropin releasing factor (CRF)-expressing neurons which project to the median eminence, the PVN has direct control over the activity within the hypothalamo-pituitary-adrenal (HPA) axis. When activated, the HPA-axis leads to the release of adrenocorticotropin releasing hormone (ACTH) in the pituitary. ACTH is responsible for the release of glucocorticoids, creating a stress response (Ferguson et al., 2008). Given the fact that the PVN is a major output of the BNST_ALG_, it is evident that the BNST is well placed for the regulation of stress-related behavior. However, in rodents, the BNST_ALG_ nuclei also project to other hypothalamic regions, including lateral hypothalamus (LH), that are involved in regulating autonomic behavior (e.g., arousal) (Dong et al., 2001b; Dong and Swanson, 2004).

### Hippocampus

Apart from its role in episodic memory and spatial navigation (Poppenk et al., 2013), the subiculum as one of the main output regions of the hippocampus has been shown to play an important role in regulating the HPA-axis. This regulation is mediated almost exclusively via the BNST as a relay center and this connection was first described in rodents (Cullinan et al., 1993; Dong et al., 2001a), but has since also been reported in both humans and non-human primates (Berry et al., 2022).

### Thalamus

The thalamus is reciprocally connected to the BNST_ALG_. The main thalamic nuclei projecting to the BNST in rodents are the anteromedial and the paraventricular nucleus (PVT) (Sun et al., 2023; Williford et al., 2023). The anteromedial nucleus of the thalamus has reciprocal connections with many parts of the limbic system (Sun et al., 2023). The paraventricular nucleus of the thalamus is involved in the integration of threats and arousal (Williford et al., 2023), and provides glutamatergic input to the BNST (Zhao et al., 2022). In humans, connections between the BNST and centromedial nucleus of the thalamus are extensive (Avery et al., 2014) and although a direct link between the BNST and the thalamus has not yet been described in primates its existence is likely.

### Midbrain/hindbrain

Further dorsal BNST_ALG_ connections include several brain stem nuclei such as the periaqueductal gray (PAG) and the parabrachial nucleus (PBN) (Dong et al., 2000, 2001a; Dong and Swanson, 2004; Hofmann and Straube, 2019). The PAG is involved in regulating autonomic functions, such as cardiovascular, respiratory, and pain responses (Faull et al., 2019), whereas the PBN is a relay center that conveys signals from the spinal cord to the thalamus and amygdala (Cersosimo and Benarroch, 2013). BNST_ALG_ output to these brain stem nuclei is thought to modulate autonomic functions in relation to stress and anxiety (Crestani et al., 2013). The PBN sends dense glutamatergic projections to the BNST and has an activating role but also is thought to be able to regulate transcription in the BNST via release of the neuropeptide pituitary adenylate cyclase-activating polypeptide (PACAP). Activation of the ovBNST via the PBN has been shown to produce anxiety like behavior in mice (Boucher et al., 2022). BNST connections to and from these autonomic brain stem centers have also been found in both rodents and humans (Dong et al., 2001a; Hofmann and Straube, 2019; Sun et al., 2023), with evidence still lacking in non-human primates. As the BNST plays an important role in reward and addiction (Volkow et al., 2019), its connection to the midbrain ventral tegmental area (VTA) and NAc and the dopamine reward system has also been extensively studied in rodents, primate and humans (Kudo et al., 2012; Kaufling et al., 2017; Miura et al., 2022). Dopaminergic VTA neurons project to the medial prefrontal cortex (mPFC) and the NAc which in resting state are thought to be mostly silent due to inhibition from gamma-aminobutyric acid (GABA-)ergic inputs from the ventral pallidum (Kudo et al., 2012). The BNST_ALG_ also provides GABAergic output to both the NAc and the ventral pallidum and activity in the BNST_ALG_ can lead to disinhibition of VTA neurons (Dong et al., 2000, 2001a; Dong and Swanson, 2004). Interestingly, BNST output to the VTA does not appear to be equal in all species. For example, one study showed that although mice have a larger absolute population of BNST neurons projecting to the VTA, they tend to make significantly lower numbers of synaptic contacts with neurons in the VTA as compared to rats, which can be interpreted as BNST_ALG_ having an overall reduced regulatory influence on the mouse VTA as compared to rats (Kaufling et al., 2017). A direct reciprocal connection between dopaminergic VTA neurons and the BNST has also been shown to be present in non-human primates (Fudge and Haber, 2001), but in general midbrain/hindbrain connections with the BNST have not been extensively studied in primates and humans. Indeed, this BNST-VTA connections has not been confirmed in humans and remains the only reported midbrain/hindbrain connection in non-human primates (Fudge and Haber, 2001).

### Basal ganglia

As mentioned above, the NAc as part of the dopamine reward system, is reciprocally connected with the BNST_ALG._ and studied extensively in rodents (Dong et al., 2000, 2001a; Dong and Swanson, 2004; Kaufling et al., 2017). However, the BSNT_ALG_ has also been shown to target other nuclei of the basal ganglia, including the striatum and substantia nigra (Smith et al., 2016). Using a variety of techniques, it was shown that these connections were functional and consisted of mainly inhibitory GABAergic projections to striatum (Smith et al., 2016) and both GABAergic and glutamatergic projections to substantia nigra pars compacta (SNc). So far, such connections have not been investigated in rats or humans or non-human primates in as much detail. However, in humans a direct and strong connection between the BNST and dorsal striatum (i.e., caudate) has been revealed by both fMRI and diffusion tensor imaging (DTI) (Avery et al., 2014; Torrisi et al., 2015) suggesting that the BNST might significantly impact and regulate the activity of the basal ganglia in many species.

Overall, the input-output connectivity of the BNST is highly complex and our current understanding is largely based on studies in rodents. As mentioned above, primate and human BNST functional connectivity has been researched in less detail partly due to limitations to the resolving power of the techniques that can be employed. However, general afferents to the anterior BNST in primates and humans do appear to largely overlap with those seen in rodents, suggesting that the major inputs and outputs of the anterior BNST_ALG_ are similar between species, notwithstanding several exceptions as outlined above. Dense innervation to the BNST include those from the PFC, CeA/MeA, subiculum, PVT of the thalamus and hypothalamus, and brainstem autonomic centers. Interestingly, compared to rodent studies, humans seem to have more and stronger connectivity between the BNST, the PFC and the basal ganglia and particularly the caudate nucleus (Fudge and Haber, 2001; Avery et al., 2014; Torrisi et al., 2015).

## Electrophysiological and morphological characterization of BNST neurons

### GABAergic and glutamatergic neurons

Anatomical and functional connectivity studies in rats, mice, primates, and humans so far have revealed both common pathways but also several differences amongst species. In humans, functional imaging of the BNST subnuclei remains a challenge, and resolving the functional contributions of selective cell types has not been possible so far. For this reason, animal models, and in particular rodents, have been essential to disentangle the roles of specific cell types within the BNST. Neuroanatomical characterization studies in rodents have suggested that glutamate and GABA are the principal excitatory and inhibitory neurotransmitters in the BNST (Table 1). Depending on further subdivisions, the relative number of GABAergic and glutamatergic neurons varies, with the vBNST containing the largest population of glutamatergic neurons (Poulin et al., 2009). In the BNST_ALG_, the vast majority of around 80%, of neurons, can be classified as GABAergic projection neurons and local interneurons, whereas only a minority of neurons, around 20%, are classified as glutamatergic projection neurons (McDonald, 1983; Sun and Cassell, 1993; Hammack et al., 2007; Nguyen et al., 2016; Daniel et al., 2017) as defined using differential expression of specific markers; glutamatergic neurons express VGLUT2 (vesicular glutamate transporter 2), whilst GABAergic neurons express VGAT and GAD65 (vesicular GABA transporter and 65 kDa isoform of glutamic acid decarboxylase, respectively). However, within these broad populations of GABAergic and glutamatergic neurons in the BNST exist a multitude of distinct neuronal subpopulations further characterized by differences in electrophysiology, morphology, neuropeptide profile, and cell-type-specific receptor expression.

### Electrophysiological subtypes: *Type I*, *Type II*, and *Type III* neurons

**FIGURE 2:**
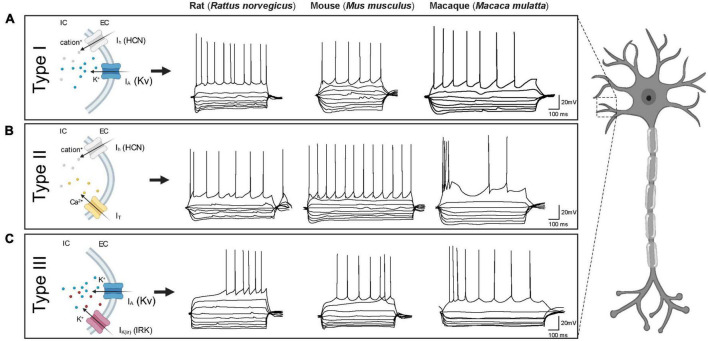
Electrophysiological characteristics of BNST neurons in three different species (rat, mouse and macaque). Each BNST neuron type displays a unique firing pattern due to the presence of certain combinations of ionic currents. **(A)** Type I neurons display similar firing characteristics across species: a regular firing pattern with depolarizing current steps and a prominent sag with hyperpolarizing current steps. **(B)** Type II neurons in both rats and macaques, but not mice, exhibit burst firing. Note the prominent rebound spike/burst after hyperpolarization in rats which is not evident in macaques. In mice on the other hand, certain Type II neurons display rebound firing while others do not (e.g., in specific trace shown here). **(C)** Type III neurons in all three species display a delay in action potential firing with depolarizing current steps and a prominent inward rectification with hyperpolarization current steps. Traces adapted from Daniel et al. (2017) with kind permission. I_h_ = hyperpolarization-activated non-specific cation current, I_T_ = low-threshold calcium (Ca^2+^) current, I_A_ = transient voltage-dependent potassium (K^+^) current, I_K(IR)_ = inward rectifying potassium (K^+^) current.

The electrophysiological heterogeneity of BNST neurons has been extensively studied in rodents by multiple research groups (Hammack et al., 2007; Szücs et al., 2010; Hazra et al., 2011; Dabrowska et al., 2013; Rodríguez-Sierra et al., 2013; Silberman et al., 2013; Nagano et al., 2015; Daniel et al., 2017; Yamauchi et al., 2018). Historically defined in the BNST_ALG_ of adult male rats by Hammack and others, three GABAergic neuronal cell types (*Type I, II, and III*) have been described based on their responses to hyperpolarizing and depolarizing current injections. These responses are shaped by the presence of four key membrane currents: (*1*) the hyperpolarization-activated non-specific cation current (I_h_), (*2*) the low-threshold calcium (Ca^2+^) current (I_T_), (*3*) the transient voltage-dependent potassium (K^+^) current (I_A_), and (*4*) the inward rectifying potassium (K^+^) current I_K(IR)_ (Figure 2). These currents are the result of the expression of four distinct ion channel types. All these channels consist of multimeric proteins, which can be comprised of more than one pore-forming subunit, leading to additional diversification of the neuronal population (Nichols and Lopatin, 1997; Randall and Tsien, 1997; Robinson and Siegelbaum, 2003; Varga et al., 2004; Hazra et al., 2011). Although *Type I*, *Type II*, and *Type III* neurons have originally been described in the BNST_ALG_, the same cell types can also be found in other subnuclei of the BNST, including the amBNST and vBNST (Rodríguez-Sierra et al., 2013). Moreover, the same classification system has also been appropriated for describing neuronal cell types in the BNST of other animal species such as mouse and macaque (Daniel et al., 2017). However, there appear to be some significant differences in the relative numbers of these cell types as well as their electrophysiological and morphological properties (see Table 2). To date, only one cross-comparison study by Daniel et al. (2017) has investigated the electrophysiological differences between several species and has suggested both commonalities and differences which provides an excellent basis for future investigations.

**TABLE 2:** Overview of the physiological and morphological properties of BNST_ALG_ neurons in rat, mouse and macaque.

	Rat (*Rattus norvegicus*)				Mouse (*Mus musculus*)				Macaque (*Macaca mulatta*)			
	***Type I***	***Type II***	***Type III***	***Other***	***Type I***	***Type II***	***Type III***	***Other***	***Type I***	***Type II***	***Type III***	***Other***
Incidence (%)	40–66% (Hammack et al., 2007; Rodríguez-Sierra et al., 2013; Daniel et al., 2017)	11–30% (Hammack et al., 2007; Rodríguez-Sierra et al., 2013; Daniel et al., 2017)	16–30% (Hammack et al., 2007; Rodríguez-Sierra et al., 2013; Daniel et al., 2017)	4–8% (Rodríguez-Sierra et al., 2013)	13% (Daniel et al., 2017)	22% (Daniel et al., 2017)	54% (Daniel et al., 2017)	11% (Daniel et al., 2017)	3% (Daniel et al., 2017)	16% (Daniel et al., 2017)	56% (Daniel et al., 2017)	25% (Daniel et al., 2017)
Spiking/firing pattern	RF,DF (Hammack et al., 2007; Rodríguez-Sierra et al., 2013; Daniel et al., 2017)	LTB, RBF (Hammack et al., 2007; Rodríguez-Sierra et al., 2013; Daniel et al., 2017)	RF, DF, fIR (Hammack et al., 2007; Rodríguez-Sierra et al., 2013; Daniel et al., 2017)	LF (Rodríguez-Sierra et al., 2013) SA (Rodríguez-Sierra et al., 2013)	RF,DF (Daniel et al., 2017; Walter et al., 2018; Miura et al., 2023)	noRBF (Daniel et al., 2017) RBF (Walter et al., 2018; Miura et al., 2023)	RF, DF, fIR (Daniel et al., 2017; Walter et al., 2018; Miura et al., 2023)	fAHP (Daniel et al., 2017)	RF,DF (Daniel et al., 2017)	LTB, RBF (Daniel et al., 2017)	DF, fIR (Daniel et al., 2017)	fAHP (Daniel et al., 2017) SF (Daniel et al., 2017)
Neuro- transmitter	GABA (McDonald, 1983; Sun and Cassell, 1993; Hammack et al., 2007; Nguyen et al., 2016; Daniel et al., 2017)	GABA (McDonald, 1983; Sun and Cassell, 1993; Hammack et al., 2007; Nguyen et al., 2016; Daniel et al., 2017)	GABA (McDonald, 1983; Sun and Cassell, 1993; Hammack et al., 2007; Nguyen et al., 2016; Daniel et al., 2017)	GABA and GLUT? (McDonald, 1983; Sun and Cassell, 1993; Hammack et al., 2007; Nguyen et al., 2016; Daniel et al., 2017)	GABA (Daniel et al., 2017)	GABA (Daniel et al., 2017)	GABA (Daniel et al., 2017)	GABA and GLUT? (Daniel et al., 2017)	GABA (Daniel et al., 2017)	GABA (Daniel et al., 2017)	GABA (Daniel et al., 2017)	GABA and GLUT? (Daniel et al., 2017)
Ion channel expression	I_h:_ HCN2 and HCN3 (Hammack et al., 2007; Hazra et al., 2011; Rodríguez-Sierra et al., 2013; Daniel et al., 2017) I_A_ (Hammack et al., 2007; Hazra et al., 2011; Rodríguez-Sierra et al., 2013; Daniel et al., 2017)	I_h,:_ HCN1-HCN4 (Hammack et al., 2007; Hazra et al., 2011; Rodríguez-Sierra et al., 2013; Daniel et al., 2017) I_T_: CaV3.1-3.3 (Hammack et al., 2007; Hazra et al., 2011; Rodríguez-Sierra et al., 2013; Daniel et al., 2017)	I_K(IR)_: Kir2.1-Kir2.4 (Hammack et al., 2007; Hazra et al., 2011; Rodríguez-Sierra et al., 2013; Daniel et al., 2017) I_A_ (Hammack et al., 2007; Hazra et al., 2011; Rodríguez-Sierra et al., 2013; Daniel et al., 2017)	I_D_ (Rodríguez-Sierra et al., 2013)	I_h_ (Daniel et al., 2017) I_A_ (Hammack et al., 2007; Hazra et al., 2011; Rodríguez-Sierra et al., 2013; Daniel et al., 2017)	I_h_ (Daniel et al., 2017) I_T_ (Daniel et al., 2017)	I_K(IR)_ (Daniel et al., 2017) I_A_ (Daniel et al., 2017)	?	I_h_ (Daniel et al., 2017)	I_h_ (Daniel et al., 2017) I_T_ (Daniel et al., 2017)	I_K(IR)_ (Daniel et al., 2017)	?
Neuropeptide markers		Enk, PKCδ (Daniel and Rainnie, 2016) NPY, SOM (McDonald and Zaric, 2015)	CRF (Dabrowska et al., 2013)	?	NPY (Walter et al., 2018)	Enk, PKCδ (Daniel and Rainnie, 2016) NPY (Walter et al., 2018)	CRF (Martin et al., 2010) NPY (Walter et al., 2018)	?	?	?	?	?
Morphology	Medium-spiny-like, round, polygonal, fusiform (McDonald, 1983; Larriva-Sahd, 2006)				Medium-spiny-like (Daniel et al., 2017)				Medium-spiny-like (Fudge and Haber, 2001) Long dendrites, large arborization (Daniel et al., 2017)			

RF, regular firing; DF, delayed firing; LTB, low-threshold bursting; RBF, rebound firing; fIR, fast-inward rectification; LF, late firing; SA, spontaneous activity; fAhP, fast afterhyperpolarization; SF, stutter-firing; GABA, gamma-aminobutyric acid; GLUT, glutamate; CRF, corticotropin releasing factor; ENK, enkephalin; NPY, neuropeptide Y; PKCδ, protein kinase C type delta.

#### Type I neurons

Originally, *Type I* neurons were first described in rats as neurons that display a fast onset sag, indicative of an I_h_ current, in response to hyperpolarizing current injections. When depolarized, *Type I* neurons typically exhibit a steady firing rate (regular firing) and a delay in action potential initiation due to a transient voltage-dependent potassium (K^+^) current (I_A_) (Hammack et al., 2007; Hazra et al., 2011; Rodríguez-Sierra et al., 2013). Using single cell reverse transcriptase polymerase chain reaction (scRT-PCR), Hazra et al. (2011) were able to demonstrate that the I_h_ channel subunits in rat BNST_ALG_ neurons are encoded by four genes, HCN1-HCN4, with each subunit differing in its activation and inactivation kinetics. Depending on the specific BNST_ALG_ neuron type these subunit isoforms are differentially expressed. For example, in the case of *Type I* neurons, only HCN2 and HCN3 subunits are expressed (Hazra et al., 2011). Although neurons that meet the original electrophysiological description of *Type I* neurons have been found in the BNST_ALG_ of both mouse and primate, the relative proportion classified as *Type I* in these species is thought to be much lower (mouse: 13%; rhesus monkey: 3%) as compared to rats (rats: 40–66%; Table 2). However, when looking at electrophysiological properties between species, there are no significant differences between rats, mice, and non-human primates (Daniel et al., 2017).

#### Type II neurons

*Type II* neurons are recognized by their burst firing activity and/or a prominent low-threshold depolarizing wave, indicative of an I_T_ current, in response to depolarizing current steps (Rodríguez-Sierra et al., 2013; Hammack et al., 2021). ScRT-PCR revealed that this I_T_ current is mediated by the presence of calcium (Ca^2+^) channels encoded by the Cav3 genes (CaV3.1-3.3) (Hazra et al., 2011). When hyperpolarized, *Type II* neurons tend to display rebound firing and a pronounced sag, indicative of a fast-activating I_h_ current. In contrast to *Type I* neurons, *Type II* neurons in rats express high levels and heterogeneous combinations of all four HCN genes. Consequently, *Type II* neurons can be split further into several subgroups (Hammack et al., 2007; Hazra et al., 2011). Looking at their overall incidence across species, in rats, 11–30% are *Type II* neurons, whilst in mice and macaques 22 and 16%, respectively, are *Type II* cells (Hazra et al., 2011; Daniel et al., 2017). Moreover, other species-specific differences have been noted in the electrophysiological properties, namely in the I_h_ and I_T_ currents which result in different firing patterns. In primates, less than half of the *Type II* cells exhibit any indication of an I_h_ current (Daniel et al., 2017). Furthermore, in comparison with rats, the I_h_ current in mice and primates is less pronounced, potentially indicating differences in subunit composition or channel kinetics (Daniel et al., 2017). In addition to the variable size of the I_h_ current, the I_T_ current also varies between species (Daniel et al., 2017). Due to variations in these ionic currents, the firing pattern of BNST_ALG_ *Type II* neurons are not uniform across species and within species, hindering robust classification based solely on their electrophysiological characteristics. For example, in contrast to rats, the firing pattern of *Type II* neurons in rhesus macaques displays a prominent burst firing, but no rebound firing (Figure 2; Daniel et al., 2017). On the other hand, in mice, *Type II* neurons exhibit rebound firing, but no burst firing pattern (Daniel et al., 2017), although also here there is quite some heterogeneity (Walter et al., 2018; Miura et al., 2023). As the BNST is known to be sexually dimorphic, one would expect that female-male differences might also be present in the electrophysiological characteristics of BNST neurons. A study in mice by Smithers et al. (2019) examined the effect of animal sex on the electrophysiological properties and excitability of *Type I* and *Type II* cells in the BNST_ALG_. Interestingly, female *Type II* neurons showed less excitability as compared to males. These sex-specific differences in excitability may potentially contribute to altered susceptibility to anxiety-related disorders (Smithers et al., 2019).

#### Type III neurons

*Type III* neurons do not exhibit a prominent I_h_ or I_T_ current, but instead display a pronounced fast rectification in response to hyperpolarizing current injections, indicative of an inwardly rectifying potassium (K^+^) current [I_K(IR)_] (Rodríguez-Sierra et al., 2013; Hammack et al., 2021). These I_K(IR)_ channels are encoded by Kir2.1-Kir2.4 genes (Hazra et al., 2011). As in rat *Type I* neurons, *Type III* neurons have a regular firing pattern when depolarized but in addition exhibit a delay in action potential initiation due to the presence of an I_A_ current. Interestingly, in contrast to rat BNST_ALG_ neurons, the most common BNST_ALG_ cell type in both macaque and mouse is *Type III* (rat: 16–30%; mouse: 54%; macaque: 56%) (Daniel et al., 2017). Although the electrophysiological phenotype of *Type III* neurons in the three species at first glance look quite similar, there are some notable differences. In particular, the spiking pattern in primates and mice differs from those defined in rats (Figure 2). Specifically, the latency to the first spike is significantly shorter in mice as compared to rats and macaques, which may be partly due to variations in the levels of I_A_ current. Due to these variations, the threshold for action potential generation is higher in primates and rats as compared to mice (Daniel et al., 2017).

#### Other neurons

Even though all three GABAergic cell types can be found in the BNST_ALG_ of rodents and primates, many other cells that do not fit any of these three electrophysiological phenotypes are also observed (Daniel and Rainnie, 2016; Daniel et al., 2019). In rodents, the vast majority of BNST_ALG_ neurons can be classified in one of three physiologically defined cell types, whilst in macaques one fourth of all BNST_ALG_ neurons do not fit in this classification scheme. Nonetheless, in rats, late firing (LF) neurons that display a conspicuous delay in action potential firing due to a slow inactivating potassium current (I_D_) in response to suprathreshold depolarizations have also been observed which do not fit in any of the three electrophysiological phenotypes (Rodríguez-Sierra et al., 2013). Additionally, neurons with high spontaneous activity (SA) at rest have also been found in the BNST_ALG_ (Rodríguez-Sierra et al., 2013). Whole cell patch-clamp recordings in both mice and macaques have identified neurons with a strong fast-inward rectification [I_K(IR)_] and a small, slow depolarizing sag (I_h_) in response to hyperpolarizing current injections. In mice, this combination of ion channels results in a unique action potential firing pattern, described as regular spiking with frequency adaptation and a large fast afterhyperpolarization (fAHP). In macaques, a similar physiological phenotype is present. However, these primate fAHP neurons have a slower firing rate as compared to mouse fAHP neurons (Daniel et al., 2017). Interestingly, another group of macaque BNST_ALG_ neurons display a unique stutter-firing (SF) pattern. These cells have no I_h_ current but do show strong inward rectification [I_K(IR)_] as seen in *Type III* cells. Despite their similarity, these neurons do not display a regular firing pattern (Daniel et al., 2017).

Further electrophysiological studies are clearly needed to completely capture the electrophysiological heterogeneity in the BNST_ALG_ and determine whether the neurons classified as “other,” are either GABAergic, such as *Type I-III*, or glutamatergic (Nguyen et al., 2016). Given the fact that some neuron types have very similar spiking patterns (e.g., regular spiking in *Type I* and *Type III* cells), classifications are particularly sensitive to experimental conditions and to accurately classify these cells additional information is likely needed. These could include markers for neurochemical content, combinations of whole-cell patch-clamp recordings combined with pharmacological approaches or mRNA analysis of transcripts of ion channel subunits together with morphological analysis of neurons.

### Morphology

Given the fact that the BNST_ALG_ consists of various unique electrophysiologically-defined cell types, it might be anticipated that these neurons also differ in their morphological properties. To date, no definitive correlation between morphology of BNST_ALG_ neurons and the three electrophysiological phenotypes has been described (Rodríguez-Sierra et al., 2013; Daniel et al., 2017). However, two Golgi-impregnation studies in rats have revealed an impressive array of neuronal morphologies in the different subnuclei of the BNST_ALG_ (McDonald, 1983; Larriva-Sahd, 2006). For example, in the juBNST, the majority of neurons are small with spiny and often bipolar dendritic trees. In the ovBNST, 10 additional types of neurons have been identified, including oval, fusiform and polygonal neurons and likely include both projection neurons and local interneurons (Larriva-Sahd, 2006). Remarkably, no such detailed study has yet been conducted in mice or non-human primates. In humans on the other hand, a wide variety of BNST neuron morphologies has also been noted, including fusiform, triangular, medium-sized, and small, basket-like neurons (Lesur et al., 1989). In general, the majority of BNST_ALG_ neurons appear to be similar in morphology to striatal medium spiny neurons and characterized by an ovoid soma with four to five spiny dendrites that branch several times (McDonald, 1983; Fudge and Haber, 2001; Table 2). Even though the general appearance of BNST_ALG_ neurons is similar across species, certain differences can be observed regarding their dendritic trees (Daniel et al., 2017). Most interestingly, primate BNST_ALG_ neurons have overall longer dendritic lengths and more dendritic branches as compared to rodents, indicative of a more complex dendritic arbor and suggestive of an increased receptive field, with individual neurons receiving a wider variety of input signals (Daniel et al., 2017).

Taken together, these various morphological and physiological characteristics of BNST_ALG_ neurons, combined with diverse synaptic inputs, are likely to have a significant impact on the integration, processing, and output of BNST_ALG_ nuclei. While most of the physiological BNST output signals have been identified as GABAergic or glutamatergic, co-expression of multiple neuropeptides in BNST neurons has also been found to modulate downstream target neurons adding further complexity but also providing an opportunity for their classification into specific neuronal types.

## Neurochemical characterization of BNST neurons

**FIGURE 3:**
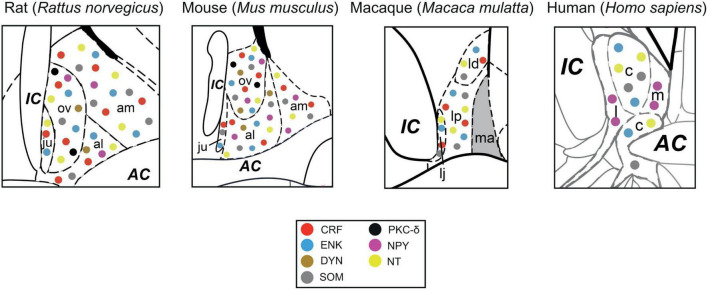
Neurochemical content of neurons in different BNST_ALG_ subnuclei of the rat, mouse, macaque and human. AC = anterior commissure, al = anterolateral BNST, am = anteromedial BNST, c = central BNST, IC = internal capsule, l = lateral BNST, ld = laterodorsal BNST, lj = lateral juxtacapsular BNST, lp = lateral posterior BNST, m = medial BNST, ma = medial anterior BNST ov = oval BNST, CRF = corticotropin releasing factor, DYN = dynorphin, ENK = enkephalin, NPY = neuropeptide Y, NT = neurotensin, PKC-delta = protein kinase C type delta, SOM = somatostatin.

Cellular studies have demonstrated that GABAergic neurons, in rodent (and in part non-human primate) BNST, contain elevated expression levels of multiple neuropeptides and enzymes, including corticotropin-releasing factor (CRF), opioid peptides enkephalin (ENK) and dynorphin (DYN), neuropeptide Y (NPY), somatostatin (SOM), neurotensin (NT), and PKCδ amongst others. Fast-acting neurotransmitters released in combination with slow-acting neuropeptides is an integral part of BNST operation through which it can shape many physiological processes and behaviors (for detailed review see Daniel and Rainnie, 2016). Even though the neuropeptidergic profiles of *Type I-III* BNST neurons have not yet been clearly and unequivocally defined, neuropeptide expression patterns are thought to differ between BNST subnuclei and BNST projections to specific target regions (Figure 3; Table 1) adding further complexity to the BNST. Below we briefly discuss the main neuropeptides in the BNST that have been linked to stress and anxiety based on abundant rodent data. For a more detailed discussion of the neuropeptides in the BNST see these excellent reviews (Kash et al., 2015; Hammack et al., 2021).

### Corticotropin-releasing factor

Corticotropin-releasing factor (CRF) is a neuropeptide consisting of 41 amino-acids and can act on two different G protein coupled receptors (GPCR): CRF receptor type 1 (CRF-R1) and CRF receptor type 2 (CRF-R2) (Bale and Vale, 2004). As the most studied neuropeptide in relation to stress and anxiety, CRF is mostly known for its role in activating the HPA-axis when released from the PVN in the hypothalamus (Vale et al., 1981). Outside of the hypothalamus, the BNST_ALG_ produces the largest amount of CRF. Interestingly, differences in CRF expression between BNST_ALG_ subnuclei have been found in rat, mice, and macaque. In particular, the ovBNST in both mice and rats and the BNSTdl in macaques contains the largest amount of CRF (Swanson et al., 1983; Ju et al., 1989; deCampo and Fudge, 2013; Nguyen et al., 2016; Ortiz-Juza et al., 2021; Fudge et al., 2022). To date, no study has been performed on post-mortem human brain tissue to study the CRF contents in the BNST. The relative incidence of CRF-expressing neurons in different species has not been extensively studied, but there are some indications that overall levels of CRF are similar in mice and rats, with only a slightly lower relative number of CRF-positive neurons in mice (Wang et al., 2011). The number of CRF-neurons does not only differ between species, but the number of CRF-expressing neurons also differs significantly between male and female rats, with females having significantly higher numbers of CRF-expressing neurons (Uchida et al., 2019).

Compared to the role of CRF in the hypothalamus, CRF in the BNST_ALG_ is not directly responsible for hormone release, but influences synaptic transmission (Gungor and Paré, 2016). In rats, when CRF is added to a bath solution during whole-cell patch clamp recordings, it significantly depolarizes *Type II* neurons in the BNST_ALG_ concomitant with an increase in input resistance, while *Type I* and *Type III* remain unaffected (Ide et al., 2013). In total, around 10% of neurons in the BNST_ALG_ express CRF. Of those 10%, the vast majority are *Type III* neurons that express CRF project mostly outside of the BNST (Martin et al., 2010; Dabrowska et al., 2011). In both rodents and macaque, these BNST_ALG_ neurons are shown to be GABAergic and glutamatergic (Nguyen et al., 2016; Fudge et al., 2022). Interestingly, CRF-expressing neurons mostly synapse with non CRF-expressing neurons (Partridge et al., 2016). It has been shown that CRF positive neurons project to multiple target regions. In rodents, some of the most prominent CRF-projection sites include the NAc, VTA, CeA, PVN, PAG, and DR amongst others. A detailed overview of the BNST_ALG_ CRF-positive output projections is given in Table 1.

As the ovBNST has the highest expression of CRF, it is thought that this nucleus can exert control over BNST output and subsequently affect behavior. One way this nucleus influences BNST_ALG_ output is via serotonergic neurons originating in the dorsal raphe projecting back to BNST_ALG_ CRF-expressing neurons. A study in mice has shown that these neurons are thought to enhance fear and anxiety via the release of serotonin, diminishing BNST_ALG_ anxiolytic output to the LH and VTA when high serotonin levels are present (Marcinkiewcz et al., 2016). When mice are exposed to repeated stress, CRF-expressing neurons have been shown to exhibit synaptic plasticity in the form of long-term potentiation (LTP), leading to changes in the connections the BNST_ALG_ has with brain regions involved in stress and anxiety. Consequently, these changes could result in chronic stress and anxiety disorders (Dabrowska et al., 2013).

### Opioid peptides: enkephalin and dynorphin

Enkephalin (ENK) and dynorphin (DYN) are opioid peptides that are derived from two different precursor molecules, proenkephalin and prodynorphin, respectively. These opioid peptides are endogenous ligands to three presynaptic GPCR, the mu- (MOR), delta- (DOR), and kappa- (KOR) receptors, coupled to G_α i_ to inhibit adenylyl cyclase/cAMP pathways. Whereas ENK preferentially binds to MOR and DOR, DYN binds to KOR (Land et al., 2008). Both peptides are known to be widely expressed throughout the CNS, including the amygdala and BNST. However, their receptors are only expressed at moderate levels in these brain structures, suggesting that these opioids might not exert a strong local modulating function (Poulin et al., 2009). Retrograde tracing combined with immunostainings in non-human primates revealed ENK-positive neurons in the BNSTju and BNSTpl, but mostly in the BNStdl. In rodents, the BNST_ALG_ contains multiple subnuclei that appear to have an elevated expression of ENK and DYN. *In situ* hybridization studies in rats and mice found that ENK expression colocalizes with neurons in the ovBNST, alBNST, and juBNST, whilst DYN expression is seen in neurons located mostly in the ovBNST and alBNST (Veening et al., 1984; Veinante and Freund-Mercier, 1997; Poulin et al., 2009). Nearly half of the neurons in the rat ovBNST express ENK. Notably, these ENK expressing ovBNST neurons co-express GAD67, but not CRF or NT (Veinante and Freund-Mercier, 1997; Day et al., 1999), which seems evident as CRF and ENK have opposing signaling mechanisms.

In the ovBNST, a subset of ENK neurons that receive dopaminergic input are stress-sensitive (Kozicz, 2002). These neurons are thought to be reciprocally connected to the amygdala. A study in rats showed that dopamine modulates transmission at the synaptic inputs onto BNST ENK-positive neurons, in this way regulating the physiological responses to stress and consequently facilitating anxiolytic responses (Krawczyk et al., 2011). Interestingly, in the BNST_ALG_ of rats, ENK mRNA has been directly linked to electrophysiologically defined *Type II* neurons, some of which co-express PKCδ (Daniel and Rainnie, 2016). Around 20% of BNST_ALG_ neurons projecting to the VTA are shown to be ENK-expressing and are exclusively GABAergic (Kudo et al., 2014). On the other hand, ENK neurons projecting to the BNST_ALG_ are seen in the CeA, the medial hypothalamus, and the PVN (Arluison et al., 1994). DYN is a potent modulator of inhibitory and excitatory synaptic transmission. Its release and signaling within the BNST has been shown to produced anxiogenic effects via KOR mechanisms (McLaughlin et al., 2003; Land et al., 2008). In mice, DYN-positive alBNST and ovBNST neurons are found to be GABAergic, acting on glutamatergic input from the BLA, which triggers anxiety-like behavior (Crowley et al., 2016). Moreover, DYN has also been shown to inhibit GABAergic synaptic transmission by acting on KOR (Li et al., 2012). Interestingly, a study in rats revealed that acute stress stimulates the release of DYN. In this regards, the effects of DYN seem to be the same as those associated with CRF (Morley et al., 1982). To date, no electrophysiological phenotype (*Type I-III*) has been linked to DYN expressing neurons.

### Neuropeptide Y

Neuropeptide Y (NPY) is a 36 amino-acid neuropeptide, is widely distributed throughout the CNS and is known to be present in the BNST_ALG_ of rodents (Tatemoto et al., 1982; Adrian et al., 1983). The peptide is generally expressed in GABAergic interneurons (Tasan et al., 2010). However, retrograde tracing in combination with immunohistochemistry in rats reported long-range NPY-positive BNST projections to the BLA (Leitermann et al., 2016). In humans, both the BNSTl and BNSTm are known to contain NPY expressing neurons (Walter et al., 1991). The peptide is known to produce anxiolytic effects, as activation of NPY in rats reduces fear potentiated startle (Gutman et al., 2008). On a cellular level, NPY is thought to be expressed in all three GABAergic electrophysiologically defined cell types (*Type I-III*), with the highest prevalence in *Type I* neurons of the BNST_ALG_ (Walter et al., 2018). These NPY positive neurons co-express SOM in rats (McDonald and Pearson, 1989; McDonald and Zaric, 2015). Furthermore, CRF and NPY are thought to bi-directionally modulate inhibitory synaptic transmission in the BNST, enhancing and inhibiting GABAergic transmission, respectively (Kash and Winder, 2006). NPY is known to significantly hyperpolarize *Type II*, but not *Type I and III* neurons in the BNST_ALG_. This hyperpolarization seen in *Type II* neurons is thought to originate due to suppression of the I_h_ current (Kash and Winder, 2006; Ide et al., 2013).

### Somatostatin

Somatostatin (SOM) is a well-known 14–28-amino acid neuropeptide that binds to GPCRs to potently inhibit adenylyl cyclase/cAMP signaling (Klimaschewski, 2009). SOM is known to be expressed in a subset of GABAergic neurons within the BNST. In rats, approximately 5–25% of the neurons in the whole BNST (anterior-posterior) and the lateral/capsular CeA express SOM (Finley et al., 1981; Shimada et al., 1989; Kiyama and Emson, 1990; Hammack et al., 2021). In rodents, the vast majority of SOM cells are located in the ovBNST and alBNST (Ju et al., 1989; Nguyen et al., 2016). Interestingly, SOM positive cell do not colocalize with PKC-delta positive cells, distinguishing two neuronal cell populations wherein SOM expressing cells constitute the main source of long-range projections to both the PAG and LPN in mice (Veinante and Freund-Mercier, 1997). Studies in non-human primates are rather limited. However, one study demonstrated that the BNST in macaques varies in its expression of SOM, depending on the specific subnucleus. The BNSTLj and BNSTlp are seen to have lower SOM levels as compared to the BNSTld (deCampo and Fudge, 2013). Even though the brain-related function of SOM regarding stress and anxiety behavior has been extensively studied, its specific function in the BNST is not yet fully determined. As an example, in the PFC and CeA SOM positive neurons are involved in driving passive fear responses, whilst CRF neurons drive active fear responses (Fadok et al., 2017; Cummings and Clem, 2020). A recent study by Bruzsik and colleagues revealed that specific SOM cells within the BNST promote fear memory formation in mice (Bruzsik et al., 2021). Another study revealed that GABAergic SOM positive projection neurons from the BNST onto NAc interneurons control anxiety-like responses (Xiao et al., 2021).

### Neurotensin

The 13-amino acid neuropeptide neurotensin (NT) is widely expressed in various brain regions, including the BNST in rodents, macaques, and humans (Kataoka et al., 1979; Alexander et al., 1989; Schroeder et al., 2019). Immunohistochemical staining in post-mortem human brain tissue showed NT positive cells are present in the BNSTc (Walter et al., 1991). In macaques, both the BNSTlp and the BNSTld contain NT neurons (deCampo and Fudge, 2013). The majority (approximately 80–90%) of the CRF positive neurons in the BNST and CeA in rats express NT, implying that CRF and NT have closely coordinated actions (Shimada et al., 1989). Similar to CRF, NT is thought to modulate GABAergic synaptic transmission. Specifically, post-synaptic depolarization has been shown to release vesicular NT and CRF that co-act to increase ovBNST inhibitory synaptic transmission, influencing stress-induced anxiety-like behavior in rats. The effect of NT on the excitability of *Type II* neurons in the rat BNST_ALG_ using whole-cell patch-clamp electrophysiology had been studied by Kaneko et al. (2021). Bath-application of NT depolarizes *Type II* BNST_ALG_ neurons, by binding to GPCR expressed in *Type II* neurons, leading to blocking of the potassium conductance and increasing non-selective cation conductance via adenylyl cyclase/cAMP mediated activities (Kaneko et al., 2021).

### Protein kinase Cδ

While not a neuropeptide, protein kinase C delta (PKCδ) is an important enzyme expressed mostly in the ovBNST of rodents, with its surrounding areas having little to no expression (Ye and Veinante, 2019). Expression of PKCδ mRNA is regulated by stress, increasing after restraint in a sex-dependent manner in mice (Fetterly et al., 2019). A recent study by Williford et al. (2023) revealed that BNST PKCδ neurons are activated by specific aversive conditions, playing a central role in risk assessment, and promoting anxiety-like behavior. Maladaptive responses to these aversive stimuli could in the long-term result in the development of disorders such as PTSD, GAD, and/or depression (Williford et al., 2023). Electrophysiologically, PKCδ expressing neurons in the ovBNST of mice are shown to have a significantly higher rheobase when compared to non-PKCδ expressing cells. PKCδ neurons in the BNST_ALG_ receive the highest input from the BLA, PVT of the thalamus and the CeA (Williford et al., 2023).

### Others

The multitude of neurochemicals expressed in different combinations can produce a great diversity. Indeed, single cell RNA studies have revealed that the BNST consists of up to 37 neuronal cell types based on their neuropeptide expression alone (Welch et al., 2019). In addition to the neuropeptides described above other neuropeptides and their respective receptors can be found in neurons of the BNST_ALG_. Among those are PACAP, oxytocin (OT), substance P (SP), neurokinin B (Tac2), vasopressin (Avp), cholecystokinin (CCK), and nociceptin (NOC) (Malsbury and McKay, 1987; Walter et al., 1991; Poulin et al., 2009; Kudo et al., 2014; Ahrens et al., 2018; Giardino et al., 2018; Zelikowsky et al., 2018; Kovner et al., 2019; Rigney et al., 2019; Rodriguez-Romaguera et al., 2020; Whylings et al., 2021). As mentioned previously, there is often consistent overlap between certain neuropeptides. For example, in rodents, a large percentage of SOM expressing neurons co-express SP, but not PKCδ (Shimada et al., 1989; Ye and Veinante, 2019) and NOC neurons often co-localize with SOM, CKK, or PKCδ, but not with CRF, ENK, or NT (Rodriguez-Romaguera et al., 2020).

It is important to note that besides the presence of multiple neuropeptides and their receptors, gonadal hormones also influence BNST neurons. Particularly neurons in the posterior BNST express markers for gonadal steroid hormones, including the androgen receptor (AR), progesterone receptor (PR), oestrogen receptors, and aromatase (Aro), the enzyme that converts androgens to oestrogens as shown in mice (Bayless and Shah, 2016). Research has shown that stress and gonadal hormones alter neuropeptide expression in the posterior BNST but also impact neuron structure and potentially neuronal functional properties in both the PFC and the hippocampus (Polston and Simerly, 2003; Farrell et al., 2015). Sexual dimorphism together with neuropeptide composition highlights the complexity of the BNST even within the same species. Future research should determine how this differential neuropeptide expression within and across species impacts BNST function.

## Conclusion

Altogether, the BNST contains a wide variety of neuronal cell populations, defined not only by their electrophysiological properties, but also by their function, input-output organization, and neurochemical content. Anatomically, the BNST is a highly complex and heterogeneous structure containing several subnuclei in rodents, which all have abundant diversity in their neuropeptide/enzyme expression and connectivity patterns. Tracer studies in rodents have aided the BNST research field in characterizing the extensive reciprocal structural connectivity patterns between the BNST and other brain regions, forming a BNST-mediated anxiety circuitry, which in general seems to be widely conserved from rodents to higher primates and humans. However, some higher functional connectivity is seen between the BNST and the prefrontal cortex, but also the dorsal striatum in humans. As the human prefrontal cortex is more developed, one could argue that there is more top-down regulation of the BNST in regulating anxiety responses. Despite these species’ differences in connectivity, similar electrophysiologically classified BNST cell types are present in both rodents and higher primates. However, certain electrophysiological properties seem to vary, leading to diverse neuronal spiking patterns. This variation in cellular properties - within the same species, but also across species - may arise not only by differences in ion channel expression but also be partly related to diverse neuropeptide expression, adding further to BNST complexity. Moreover, neuropeptide expression levels are seen to vary between sexes, highlighting the importance of using and controlling for both sexes in BNST research. Though this Review attempts to capture this complexity of the BNST it also reveals that more fundamental and translational research is needed. Therefore, as a future direction we hope that by using a combination of complementary techniques in rodents, primates, and humans and classification of its neuronal cell types, we might get closer to forming a general functional framework of the BNST in stress and anxiety which can form the basis of future studies regarding novel therapeutic approaches for stress-and anxiety related disorders.

## Author contributions

All authors listed have made a substantial, direct, and intellectual contribution to the work, and approved it for publication.
